# Mixed-methods organizational evaluation of a physical activity programme for cancer survivors in primary care

**DOI:** 10.1093/tbm/ibaf029

**Published:** 2025-07-07

**Authors:** Famke Huizinga, Nico-Derk Lodewijk Westerink, Annemiek M E Walenkamp, Annette J Berendsen, Marjolein Y Berger, Daan Brandenbarg

**Affiliations:** Department of Primary and Long-term Care, University Medical Center Groningen, University of Groningen, PO Box 196, FA 21, 9700 AD, Groningen, Netherlands; Department of Primary and Long-term Care, University Medical Center Groningen, University of Groningen, PO Box 196, FA 21, 9700 AD, Groningen, Netherlands; Department of Medical Oncology, University Medical Center Groningen, University of Groningen, PO Box 30.001, DA 11, 9700 RB, Groningen, Netherlands; Department of Primary and Long-term Care, University Medical Center Groningen, University of Groningen, PO Box 196, FA 21, 9700 AD, Groningen, Netherlands; Department of Primary and Long-term Care, University Medical Center Groningen, University of Groningen, PO Box 196, FA 21, 9700 AD, Groningen, Netherlands; Department of Primary and Long-term Care, University Medical Center Groningen, University of Groningen, PO Box 196, FA 21, 9700 AD, Groningen, Netherlands

**Keywords:** cancer survivorship, physical activity, primary care, implementation science, process evaluation

## Abstract

**Background:**

Physical activity (PA) has proven health benefits for cancer survivors, yet PA programmes are not routinely available in general practice.

**Purpose:**

This mixed-methods study used the *RE-AIM* framework to evaluate the *Adoption*, *Implementation*, and *Maintenance* of a PA programme at an organisational level for cancer survivors in Dutch general practice.

**Methods:**

Primary care practitioners (practice nurses, dieticians, and doctor’s assistants) delivering a PA programme aimed at increasing PA in daily activities, and general practitioners (GPs) in whose practices it was performed, completed questionnaires and interviews. Quantitative and qualitative data were analysed descriptively or by thematic analysis, respectively.

**Results:**

Concerning *Adoption*, 9% of general practices (*n* = 14) took part and showed high representativeness. Primary care practitioners coached a median of seven patients over 18.5 months, with barriers and facilitators emerging mainly related to organizational support, programme alignment, and patient health benefits. Concerning *Implementation*, adherence to the protocol was 77%, and the training was evaluated as 8 out of 10. Concerning *Maintenance*, 11 primary care practitioners (69%) used programme elements outside the study context.

**Conclusions:**

We conclude that our PA programme seems feasible in general practice provided there is sufficient organizational capacity. Designating a lead-motivated practitioner, providing sufficient training, and aligning and integrating PA counselling in routine care are key to providing appropriate and targeted support for cancer survivors in general practice.

## Teaser

Physical activity programmes for survivors of cancer can be feasibly offered in primary care as a proof of principle, provided they align with regular care, are led by a motivated trained primary care practitioner, and have adequate time, budget, and personnel.

## Graphical Abstract

**Graphical Abstract:**
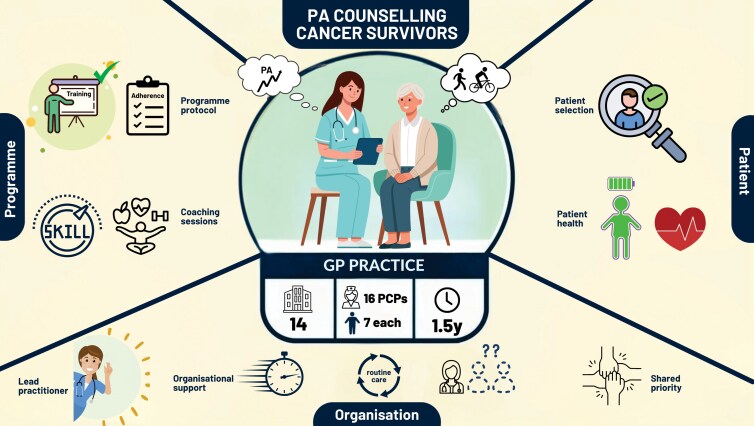

**Implications**

**Practice**: Physical activity programs for cancer survivors can be implemented in primary care when there is sufficient organizational capacity, with integration into routine care and the involvement of a trained primary care practitioner.

**Policy:** National implementation of physical activity programs for cancer survivors in primary care requires policy and insurance level changes with the allocation of sufficient time, budget, and workforce.

**Research:** Future implementation research should be aimed at the development, implementation, and evaluation of specific guidelines for physical activity promotion among cancer survivors with clear demarcation of roles for primary care practitioners and other health care workers involved.

## Introduction

Physical activity (PA) improves cancer survivors’ health [1–8], and as such has been promoted in clinical guidelines since 2010 [9, 10]. Despite this, most cancer survivors do not meet recommended PA levels, with marked differences noted between patient groups [11–13]. For example, survivors with low comorbidity, a university degree, and an active lifestyle before diagnosis are most likely to meet recommended levels [11]. Cancer- and treatment-related and organisational barriers to PA exist, including poor health, pain, fatigue, and programme accessibility in terms of cost and location [14–16]. Research indicates that successful PA programmes are adapted to the patient’s needs, are organised close to their home, and offer feedback and coaching [16, 17].

In countries with strong primary care, such as the Netherlands, primary care practitioners, general practitioners (GPs), and practice nurses (PNs) in particular, are in an ideal position to discuss or provide lifestyle care for cancer survivors [18]. GPs may have long-term relationships with patients that are built on trust and are typically accessible to patients in terms of costs and location. Survivors show increased consultation rates with their GP during and after cancer treatment and appreciate the role of GPs when discussing lifestyle issues [19, 20]. Moreover, in 80% of Dutch general practices, the PN already provides lifestyle care to patients with chronic illnesses, including diabetes, pulmonary diseases, and cardiovascular risk management [21]. However, this care differs between practices and is often not extensive and protocolized. Primary healthcare practitioners recognize the benefits of lifestyle programs like exercise and diet [22], but the integration of lifestyle care can be limited by a lack of structured programs. Currently, no PA programmes exist for cancer survivors in Dutch general practice. This represents a missed opportunity to provide accessible (both in terms of cost and location) support to a large group of cancer survivors in adopting a healthy lifestyle. Such programmes could help alleviate disease- and treatment-related complaints, potentially reducing the risk of prolonged symptoms and avoiding unnecessary referrals to secondary care. Furthermore, few studies have explored the views of primary care practitioners regarding the implementation of such programmes in these settings [22, 23].

In the SoDA study, a PA programme for cancer survivors in Dutch general practice is implemented [24]. The present research evaluates implementation at an organisational level, using a mixed-methods approach.

## Methods

### Study design and setting

The SoDA study was a single-arm implementation study of a hybrid type 1 design [25] in which a PA programme for cancer survivors was implemented in general practices in the North of the Netherlands [24]. Briefly, the programme was implemented from December 2020 to June 2024 and comprised six 30-minute in-person counselling sessions (with the possibility of phone sessions for sessions 2, 3, and 5) over 9 months with a primary care practitioner employed in general practice (i.e. PN, dietician, or doctor’s assistant). They coached survivors to increase their PA in daily activities, (i.e. walking, cycling, swimming, or gardening), using an activity tracker for goal setting and feedback. The present study evaluates implementation at an organisational level only, using the *Adoption* (proportion and representativeness of settings that adopt the program), *Implementation* (program delivery and adherence), and *Maintenance* (program’s institutionalization in routine care) domains of the RE-AIM framework [26, 27]. Both quantitative and qualitative methods were used. Evaluation at the patient level has been reported elsewhere [28, 29].

### General practice recruitment

A list of potentially interested practices was created using a regional academic GP network in the North of the Netherlands (AHON) [30] and practices known to the project team. Recruitment was by mail, telephone, social media, email, or personal contact from December 2020 to February 2023. General practices were recruited consecutively to implement lessons learned in subsequent practices.

### Procedures

When a practice agreed to participate, primary care practitioners were trained by experienced instructors (a human movement scientist and FH) in online groups of 3–5 during the COVID pandemic or individually at their practice thereafter. GPs were not trained as they did not deliver the PA programme.

Training was based on the COACH method [24], which included various motivational techniques and their theoretical underpinnings, concrete instructions on what to discuss, and information about the activity tracker. The first two sessions were practiced in peer role play, with instructors providing feedback. Oncology nurses provided training about common physical and psychosocial problems and subsequent referral options.

A list of cancer survivors was obtained from medical records, using the International Classification of Primary Care codes for cancer diagnoses. A GP or primary care practitioner then selected eligible patients as those aged ≥18 years old who had finished primary cancer treatment at least 6 months before inclusion (adjuvant hormonal therapy was allowed). Exclusion criteria were non-melanoma skin cancer, participation in another PA programme, terminal illness, physical or cognitive impairment, and any other reason judged by the GP or primary care practitioner. The GP or primary care practitioner then invited patients by telephone, letter, or during consultations.

### Support during implementation

The research team comprised a doctoral student, research assistant, clinical epidemiologist, human movement scientist, PN, medical oncologist, three GPs, and two patient advocates. Support to primary care practitioners was provided by the doctoral student and research assistant by email, telephone, or in-person visits to the general practice, and mainly related to starting and continuing implementation, such as identifying eligible patients and providing digital support with the activity tracker and data collection system. We distributed a newsletter every 3 months to increase enthusiasm and involvement, and organised peer support sessions for primary care practitioners.

### Data collection

Table 1 summarizes the RE-AIM dimensions, measures, and measurement instruments.

**Table 1:** The RE-AIM dimensions and measurement instruments

RE-AIM dimension	Measures	Measurement instrument	Time
Adoption	1.Participation rate among general practices	1.Researcher logged	T0
	2.Reasons for general practices not participating	*2.*Researcher logged	T0
	3.Characteristics and representativeness of participating general practices	3.General practice demographic questionnaire, Nivel registration Dutch general practices	T0
Implementation	1.Implementation characteristics: duration, no. patients coached, reasons for drop-out	1.PNs logged data collection system	T3
	2.Evaluation training PCPs	2.Evaluation questionnaire, *interviews PCPs*	T0
	3.Programme protocol adherence and experience	3*.Observation checklist, interviews PCPs*	S1–S6, T1–T3
	4.Use of phone sessions	4*.*PCPs logged data collection system	S2/S3/S5
	5.Barriers and facilitators of implementation	5*.Interviews GPs and PCPs, researchers’ implementation field notes*	T1–T3
Maintenance	1.Use of PA programme by PCPs among patients outside the study population	1.*Maintenance questionnaire PCPs*, *interviews PCPs*	T3
	2.Advice for future implementation	2*.Interviews PCPs and GPs*	T3

PCP, primary care practitioner (i.e. a practice nurse, dietician, or doctors assistant); GP, general practitioner; PA, physical activity; S1–S6, session 1–6 with the PN (S1 = baseline; S2 = 3 weeks; S3 = 6 weeks; S4 = 3 months; S5 = 6 months; S6 = 9 months); time T0–T3 (T0 = baseline; T1 = 3 months; T2 = 6 months; T3 = 9 months). *Italic* measurement instruments include qualitative measures. This study used a mixed-methods design with the *RE-AIM* framework to evaluate the *Adoption*, *Implementation*, and *Maintenance* of a PA programme at an organizational level for cancer survivors in Dutch general practice.

#### Adoption

The participation rate was reported, and reasons for non-participation were sought by phone or email. The characteristics of each general practice were obtained by questionnaire and compared to national Nivel registrations [31].

#### Implementation

We recorded the implementation duration (from Session 1 of the first patient to Session 6 of the last patient) and number of patients coached (minimum of 2 sessions) per practice. Primary care practitioners’ training was evaluated by self-report and interview. On a 10-point scale from 1 (very poor) to 10 (excellent) primary care practitioners reported if they felt sufficiently prepared to deliver the programme, provide coaching, use the activity tracker, and report data in the data collection system. Protocol adherence was evaluated by a researcher (FH) observing two coaching sessions delivered by each primary care practitioner, using an observation checklist, and calculating a percentage of discussed talking points. Interviews with primary care practitioners were used to evaluate the programme protocol. Primary care practitioners logged whether Sessions 2, 3, and 5 took place by phone or in-person to calculate average and individual use of phone sessions.

Barriers and facilitators were assessed by focus group, individual, or duo interviews with primary care practitioners and GPs both during and after implementation. Two researchers (FH and DB) developed topic lists for these interviews in close collaboration with the research team (Supplement 1). Interviews were held iteratively, and the topic list was adapted based on earlier interviews and quantitative outcomes in each general practice (e.g. participation rate or number of patients coached). The main question of interest was: ‘What are your experiences and related barriers and facilitators of implementing the PA programme for cancer survivors in general practice?’ We asked all primary care practitioners (*n* = 16) and at least one GP to participate per practice. They each received a gift card of 20 euros for participation, of which they were not aware *a priori*. Most interviews were conducted at the practice of the participating GPs and primary care practitioners, except for the focus groups and one individual interview, which were completed online.

FH performed the interviews, either individually or in collaboration with DB or a medical student. FH and DB are both trained interviewers. Field notes were also written down after every contact with a GP or primary care practitioner and were used to explore barriers and facilitators.

#### Maintenance

Programme use beyond the study population was evaluated by questionnaire and interview. The questionnaire asked if elements of the PA programme had been used outside the study population or after the study ended if primary care practitioners used theoretical knowledge from the training, and whether they had changed their interactions with cancer survivors. Advice for future implementation of the programme was explored in the interviews.

### Data analysis

Quantitative data were analysed using IBM SPSS Statistics for Windows, Version 23 (IBM Corp., Armonk, NY) [32] calculating frequencies or medians and ranges for descriptive analyses. Interviews were audio recorded (notes were written down afterwards for three interviews), transcribed verbatim, and pseudonymized. Qualitative data and field notes were independently analysed thematically [33] in ATLAS.ti (Version 22) by two researchers (FH with DB or a medical student) using inductive coding for text segments relevant to the research question. Final coding was by consensus. Themes were subtracted by the researchers and discussed first with a GP in the research team (NDLW) and subsequently with the other members. The Consolidated Framework for Implementation Research was used to guide theme development [34].

### Ethical approval

The Medical Research Ethics Committee of the University Medical Centre Groningen, the Netherlands, concluded that the SoDA study was not subject to the Dutch Medical Research Involving Human Subjects Act (registration number: 201900586). All participants gave informed consent for the use of their data for scientific purposes. Additional signed informed consent was obtained from the participants of which the interviews were recorded.

## Results

### Adoption

**Figure 1:**
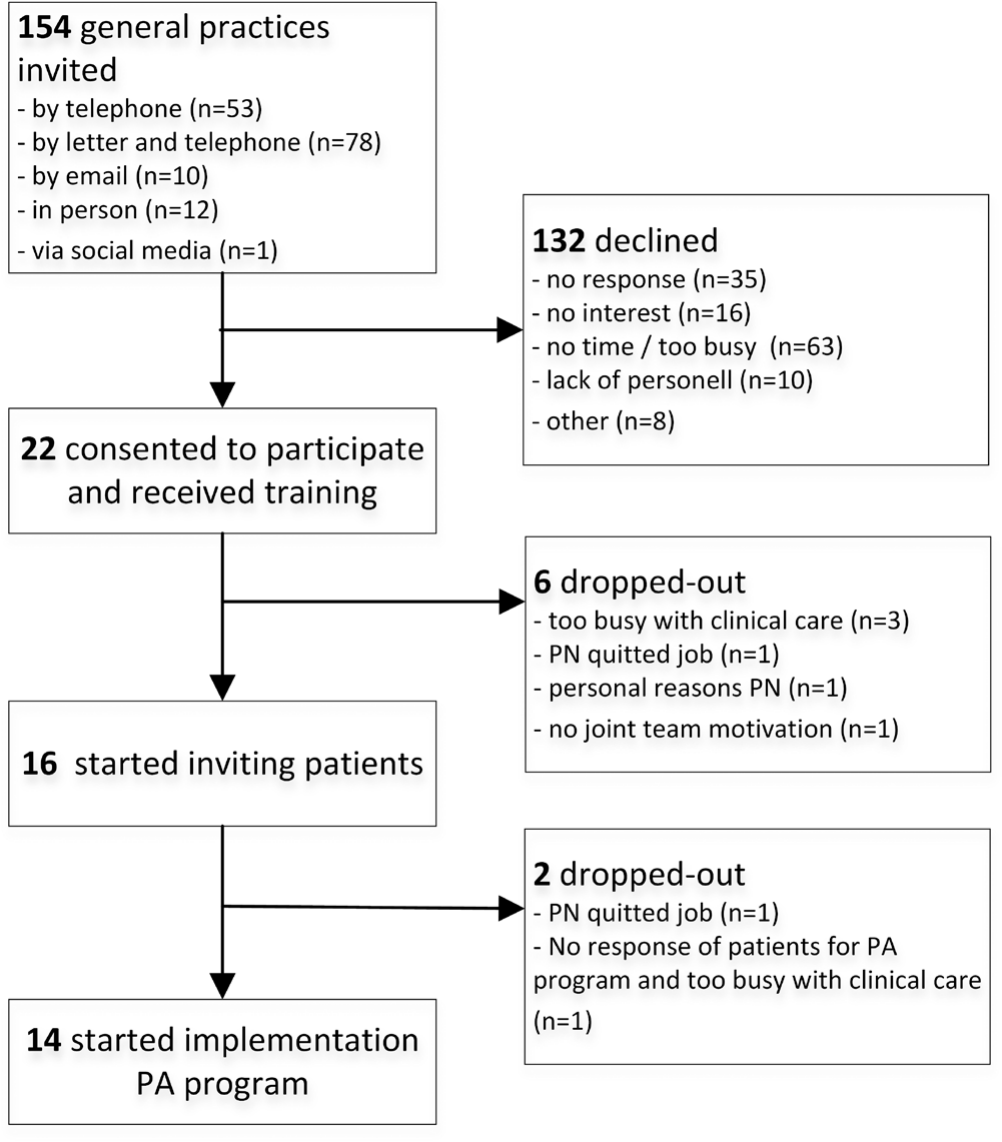
Flowchart of recruitment of general practices

Overall, 22 of 154 invited general practices agreed to participate and we trained 27 primary care practitioners, but ultimately, 14 general practices (9%) with 16 primary care practitioners started the implementation (Fig. 1). Reasons of the 132 practices that declined participation, included being too busy with clinical care (48%), lack of interest (12%), and lack of personnel (8%); 27% provided no reason.

Participating practices typically comprised 3 GPs (range: 1–10), 2 PNs (range: 0–5), and 3510 patients (range: 1316–14 919). The primary care practitioner involved in the coaching sessions was a PN in 11 general practices, a doctor’s assistant in 2, and a dietician in 1. All primary care practitioners and 55% of the GPs were female (Supplement 2), with median ages of 50 years (range: 38–63), and 47 years (range: 33–64), respectively. Median experience was 11 years for primary care practitioners (range: 2–27) and 17 years for GPs (range: 4–30).

The median number of patients per full-time GP was 1960 (range: 1535–2550; national average: 2095). Practice ownership models were solo, duo, or group in 50%, 36%, and 14%, respectively (national averages of 17%, 44%, and 39%, respectively). Most practices were in rural (50%) or urban/rural (43%) settings.

### Implementation

**Figure 2:**
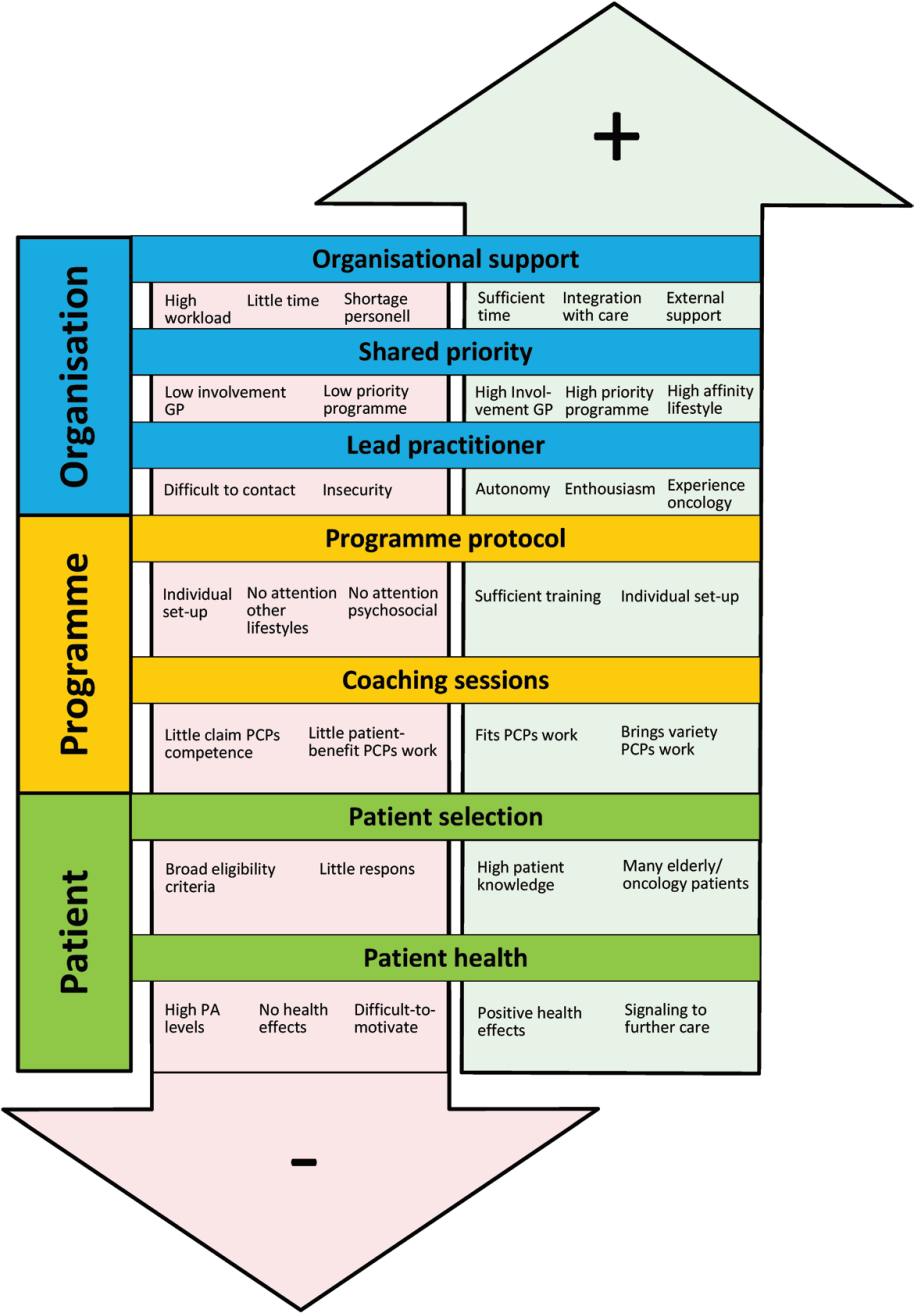
Barriers (↓; −) and facilitators (↑; +) within the domains *organisation*, *programme* and *patient*

Eleven GPs and 13 primary care practitioners were interviewed (Supplement 2). Qualitative analysis revealed three domains: *organisation*, *programme,* and *patient*. Table 2 shows the quantitative data and Fig. 2 summarizes the themes and quotes from the qualitative analysis.

**Table 2:** Implementation characteristics of participating general practices and PCPs

General practice	PCP involved in implementation	Implementation duration (months)	No patients coached#	Use of phone sessions	General evaluation training (1–10)	Adherence to protocol
1	PN1	38	28	36%	NA	80%
2	PN2	31	7	0%	8	20%
3	PN3	27	10	0%	8	75%
4	DT4	24	7	33%	9	95%
5	PN5a	24	10	61%	8	65%
	PN5b		7	0%	NA	64%
6^a^	PN6	20	13	53%	7	80%
7	PN7	20	3	20%	NA	NA
8^a^	PN8a	17	2	0%	7	NA
	PN8b		2	33%	7	NA
9	DA9	17	14	11%	9	80%
10	PN10	17	6	27%	9	85%
11^a^	PN11	15	4	17%	NA	NA
12	PN12	14	3	0%	10	80%
13	PN13	11	7	29%	8	90%
14^a^	DA14	9	3	100%	8	NA
Total, median (range) or mean		18.5 (9–38)	7 (2–28)	27%	8 (7–10)	77% (20–95)

DA, doctors assistant; DT, dietician; PCP, primary care practitioner; PN, practice nurse.

^a^Quitted with implementation before study ended; # patients who followed at least 2 sessions of the programme.

#### Organisation

Primary care practitioners coached a median of seven patients (range: 2–28) over a median duration of 18.5 months (range: 9–38) (Table 2). However, five primary care practitioners (from four general practices) dropped out before the study ended because they became ill (PN6), left their jobs (PN8b and DA14), or were too busy with clinical care (PN8a and PN11) (Table 2). Concerning *organisational support,* several primary care practitioners saw the increased workload as a barrier.

PN7: *That [workload] is the problem for me … why it doesn’t work … So, I wouldn’t know how to do it at this point. My colleague is returning, but we are now months behind.*

Use of phone instead of face-to-face sessions for Sessions 2, 3, and 5 was 29%, 37%, and 16%, respectively (average = 27%). The possibility of integrating the programme with regular care, including phone sessions, was identified as a facilitator.

PN7: *With one patient, I did it with his diabetes checks … I can actually do two in one appointment, which makes a difference … it makes it easier.*

Having sufficient time and receiving external support from researchers facilitated implementation. Other barriers related to the theme *shared priority,* which included low GP involvement and low priority in the team. By contrast, facilitation was associated with high GP involvement and a team that prioritized the programme and lifestyle factors.

GP6: *As a team you should consider it an important topic. We find it important to live a healthy lifestyle and to promote that; we are all non-smokers. You also have general practices where they all smoke […] Lifestyle has a completely different meaning within those teams.*

Other organisational factors related to the position and character of the primary care practitioner as a *lead practitioner* for PA programme delivery. While some hampered implementation by being difficult to contact or being insecure about programme delivery, an enthusiastic and motivated primary care practitioner who worked independently and had experience with oncological care facilitated implementation.

#### Programme

Twelve primary care practitioners evaluated their training from 1 (very poor) to 10 (excellent) with the following median scores: 8 (range: 7–10) for sufficient preparedness to deliver the programme; 8 (range: 7–10) for providing coaching; 6 (range: 5–10) for activity tracker use; and 8.5 (range: 5–10) for using the data collection system. Sufficient training was mentioned as a facilitator within the *programme protocol* theme. Among 11 primary care practitioners, we observed one (*n* = 1) or two (*n* = 10) sessions, noting median protocol adherence of 77% (range: 20%–95%). A barrier identified by primary care practitioners was requiring patients to perform PA individually rather than in groups. The focus on improving PA alone, with little attention to psychosocial or other lifestyle domains, was another barrier.

PN2: *It felt a bit unnatural only talking about the activity tracker […] People have come a long way, and that’s where things like fear and uncertainty come into play. It is important to pay attention to those as well; so, that’s what I did. It wasn’t in the protocol, but I thought that was my job.*

An individualized approach to both coaching sessions and PA performance was considered a facilitator, giving patients autonomy to choose their PA goals and activities.

DT4: *You don’t impose anything on them. Nothing. They decide how many and what goals they set. It was entirely up to the person … and it didn’t matter if they failed.*

Within the theme *coaching sessions*, implementation was hindered if competence was an issue; that is, if the primary care practitioner’s input was not required or if it offered little benefit to a patients’ health:

PN3: *Unfortunately, I only had people who exercise a lot […] little support was required from me.*

Implementation was facilitated if primary care practitioners felt the coaching sessions fitted in with existing lifestyle consultations as part of their usual practice, or if it brought variety through different conversations with cancer survivors as a new or unknown patient population.

#### Patient

Barriers were observed in *patient selection* concerning the broad eligibility criteria. Primary care practitioners expressed doubts about who may be suited for the programme and who to invite. Low response to invitations was experienced as another barrier. By contrast, good knowledge of their patients or working in practices with many elderly or oncology patients facilitated patient selection.

Regarding *patient health*, those who already had high PA levels without clear care needs showed no observable health benefits and were difficult-to-motivate, hindering implementation.

PN13: *My experience is that [most participants] already do a lot of exercise. […] This programme is not necessary for those people.*

By contrast, implementation was facilitated if primary care practitioners or GPs observed positive effects on patients’ health. They experienced the added value of the programme for identifying previously unidentified patients in need of further care.

GP6: *Most people achieved really good results with it […] we could see that in, for example, diabetes control: that people also had better sugars.*

### Maintenance

Of the 14 primary care practitioners who completed the maintenance questionnaire, 1 reported using the full PA programme and 11 (69%) reported using parts of the programme with patients beyond the study. This included patients with diabetes mellitus, cardiovascular disease, pulmonary disease, obesity, or cancer. The most commonly used elements were motivational interviewing, measurable goal setting and evaluation, and activity tracker use. Six primary care practitioners (43%) reported using theoretical knowledge obtained from the training in other consultations, such as the favourable effects of PA on health:

PN5a: *I gained more insight into the importance of exercise, even small improvements, and that this does not always have to be [typical] “sports.”*

Half of the primary care practitioners reported their care of cancer survivors changed after participation. They paid more attention to a history of cancer, related fatigue, and mental health issues, and to rebuilding physical health after cancer treatment.

Interviews with GPs and primary care practitioners uncovered programme-specific and general advice for future implementation. Programme-specific advice was to include the management of other lifestyle domains and psychological support, to facilitate group PA, and to offer the PA programme to patients with other diseases. Stricter eligibility criteria were also recommended, such as inclusion shortly after diagnosis (0–2 years) to reach patients with care needs. General advice for the organisation of primary care for cancer survivors included having a routine follow-up consultation or a regular care programme.

GP10: *It might be a good idea to invite patients after cancer treatment and ask: “what do you actually need from us?” You can organise something structured; for example, invite them a year after treatment ends and again a year later.*

GPs mentioned the need to avoid medicalizing cancer survivors and to seek closer collaboration with societal care, while still recognizing the need for a close partnership between primary and secondary care. They also mentioned that further implementation depended on having sufficient time, personnel, budget, and training, as well as a lead practitioner.

## Discussion

### Summary

Overall, participating primary care practitioners felt well-prepared and demonstrated good adherence to the protocol. The participation rate among general practices was low and related mainly to a lack of time, but the characteristics of the participating practices were similar to the national average. Organisational barriers and facilitators are mainly related to organisational capacity in terms of time and work force, shared team interest and responsibility for the programme, and the primary care practitioner as a lead practitioner. Implementation was facilitated by sufficient training, external support, and primary care practitioners with high independence and enthusiasm. Observing patient health benefits emerged as an important facilitator, but this was considered a barrier when primary care practitioners perceived the programme as irrelevant (e.g. if patients were already physically active and symptom-free). The use of skills and knowledge acquired by primary care practitioners during the study was high, suggesting promise for long-term maintenance. However, further implementation will require the availability of sufficient time, personnel, budget, and training.

### Strengths and limitations

To our knowledge, this is the first study that has evaluated the implementation of a PA programme for cancer survivors in general practice from the provider’s perspective. Strengths include the mixed-methods data sources, which increase the validity and reliability of our results. Also, the inclusion of representative general practices with at least one primary care practitioner interviewed per practice improves the generalizability and credibility of our findings. Limitations may include that we did not define a priori benchmarks for the quantitative measures (i.e. participation rate and adherence). Including these could have strengthened the interpretation of our findings; future implementation studies may follow-up on that. Furthermore, a low participation rate of practices limits the generalizability of our findings, especially since the most mentioned reasons for non-participation were related to workload or time constraints. Study procedures (e.g. patient measurements and data collection) may have taken extra time and may not have aligned with the primary care practitioners’ tasks. Nevertheless, our study is unique in evaluating the implementation from providers’ perspective, and we think our results will prove informative in countries with similar primary care systems, such as the UK and Denmark.

### Comparison with the literature

The low participation rate of general practices, together with the reasons for non-participation, may reflect the current high pressures on Dutch primary care due to personnel shortages, an ageing population, and the shift of care tasks from secondary to primary care. Primary care practitioners were able to coach only a limited number of patients, indicating that general practices have little capacity to deal with extra health care tasks, even with close external support. Problems go beyond the willingness and competence of staff to implement programmes, and instead, require changes at the policy and health insurance level [22]. These must ensure that time, budget, and workforce prerequisites are addressed, consistent with earlier research [22, 35, 36]. Consequently, addressing these prerequisites will be necessary if we are to implement any PA programme successfully among cancer survivors in primary care; moreover, contextual needs will differ by country and should be tailored accordingly [35].

The need to integrate PA consultations into routine care has been discussed previously and was valued by patients as well in earlier research [35, 37]. Also, providing sufficient training as a key facilitator aligns with earlier research [23]. Training is particularly important to increase oncology-specific knowledge and self-efficacy among primary care practitioners, especially because primary care practitioners may have limited experience with oncology care, lack of knowledge about cancer-related disabilities, and uncertainty regarding appropriate treatment options [23]. Where training is lacking or perceived as insufficient, this will represent a barrier to implementation [18]. Training delivered in this study was evaluated positively, and as such, may serve as an example for further research or guidelines.

Our study identified a significant barrier to implementation: the perceived lack of impact of the PA programme on patients’ health outcomes. This likely resulted from our broad eligibility criteria; as we did not select on low PA levels or symptom severity, we may have engaged a relatively healthy cohort of cancer survivors, including patients with high PA levels and no evident care needs [29]. Reaching out to this broad range of cancer survivors is also reflected in a low patient participation rate (26%), published elsewhere [29], which was experienced by primary care practitioners as a barrier as well. Fortunately, some general practices seem to have succeeded in this aspect, as our study did reach participants with less formal education and higher symptom severity, compared to the ones not participating [29]. Nevertheless, the barriers found do emphasize the need to more strictly approach only those patients who may benefit most, but identifying those remains a challenging issue. Earlier research shows that support in this selection process may be valued by primary care practitioners [38]. In our study, primary care practitioners and GPs also reflect on this issue and recommend selection shortly after cancer treatment. However, this approach may overlook individuals with long-term complaints, such as fatigue, that can persist up to 10 years after diagnosis [39, 40]. A tailored approach is therefore important [28, 35], and this requires in the first place a clear picture of both the cancer survivor population and their specific needs in primary care [35]. As suggested by GPs in this study, a routine GP–patient consultation after cancer treatment may be a good starting point to reach patients who require further care. Appropriate communication about patients discharged from secondary to primary care with sufficient notice and a clear treatment summary is therefore warranted [18, 41, 42].

Transfer of learned skills and knowledge was high among primary care practitioners. However, we measured these immediately after implementation, precluding long-term conclusions, which is a problem acknowledged in earlier research [36]. In this study, the potential to expand the PA programme to other lifestyle and psychosocial domains would be valued by primary care practitioners and patients [28]. This is consistent with the central role of general practices in preventive, lifestyle, and psychosocial care for cancer survivors [18].

### Clinical implication

To enable PA promotion among cancer survivors in general practice, we recommend that primary care practitioners gain a clear picture of the survivors in their practice and their PA-related care needs, taking the patient consultation after cancer treatment as a starting point. Depending on the specific needs of each patient, PA counselling can be provided via a lead practitioner (such as a PN) within the general practice or by seeking collaboration with other primary, secondary, or public health care providers, as recognized in this and earlier research [18, 43]. GPs may also advice patients to search for other community activities or PA programmes based in the community. If PA counselling is offered by the general practice, we recommend they designate a PA lead practitioner, to provide this lead practitioner with PA- and oncology-specific training, and to align and integrate PA counselling with routine care. Nationally, primary care practitioners may benefit from clear practical guidelines [41, 42], with particular attention on the transmission of care from secondary to primary care and a clear demarcation of roles. Active dissemination, implementation, and evaluation of these guidelines may then stimulate national integration in clinical care [44].

## Conclusion

The PA programme in this study seems feasible in primary care where there is sufficient organisational capacity. However, national implementation in Dutch general practice probably has limited feasibility due to contextual difficulties that place high pressure on the primary care system. A routine and standardized patient consultation after cancer treatment may offer a feasible starting point for gaining a clear picture of the cancer survivor population and for selecting patients who may benefit from PA or lifestyle care. Designating a lead practitioner, providing sufficient training, and aligning and integrating PA counselling in routine care will be key to providing appropriate and targeted support in general practice.

## Supplementary Material

ibaf029_suppl_Supplementary

## Data Availability

Quantitative data are available from the corresponding author on reasonable request. Qualitative data from this study are not available in a public archive or available from the corresponding author on reasonable request because of the impossibility to de-identify the data.

## Welfare of Animals

This article does not contain any studies with animals performed by any of the authors.

## References

[1] Jones LW, Liang Y, Pituskin EN, et al Effect of exercise training on peak oxygen consumption in patients with cancer: a meta-analysis. Oncologist 2011;16:112–20. https://doi.org/10.1634/theoncologist.2010-0197 21212429 PMC3228052

[2] Segal R, Zwaal C, Green E, et al; Exercise for People with Cancer Guideline Development Group. Exercise for people with cancer: a systematic review. Curr Oncol 2017;24:e290–315. https://doi.org/10.3747/co.24.3619 28874900 PMC5576469

[3] Buffart LM, Kalter J, Sweegers MG, et al Effects and moderators of exercise on quality of life and physical function in patients with cancer: an individual patient data meta-analysis of 34 RCTs. Cancer Treat Rev 2017;52:91–104. https://doi.org/10.1016/j.ctrv.2016.11.010 28006694

[4] Mishra SI, Scherer RW, Snyder C, et al Are exercise programs effective for improving health-related quality of life among cancer survivors? A systematic review and meta-analysis. Oncol Nurs Forum 2014;41:E326–42. https://doi.org/10.1188/14.ONF.E326-E342 25355029 PMC4332787

[5] Swartz MC, Lewis ZH, Lyons EJ, et al Effect of home- and community-based physical activity interventions on physical function among cancer survivors: a systematic review and meta-analysis. Arch Phys Med Rehabil 2017;1652:65. 10.1016/j.apmr.2017.03.017 PMC5534187 28427925

[6] Cramp F, Byron-Daniel J. Exercise for the management of cancer-related fatigue in adults. Cochrane Database Syst Rev 2012;11:CD006145. https://doi.org/10.1002/14651858.CD006145.pub3 23152233 PMC8480137

[7] Huizinga F, Westerink N-DL, Berendsen AJ, et al Home-based physical activity to alleviate fatigue in cancer survivors. Med Sci Sports Exerc 2021;53:2661–74. https://doi.org/10.1249/mss.0000000000002735 34649267 PMC8594505

[8] Mustian KM, Alfano CM, Heckler C, et al Comparison of pharmaceutical, psychological, and exercise treatments for cancer-related fatigue: a meta-analysis. JAMA Oncol 2017;3:961–8. https://doi.org/10.1001/jamaoncol.2016.6914 28253393 PMC5557289

[9] Campbell KL, Winters-Stone KM, Wiskemann J, et al Exercise guidelines for cancer survivors: consensus statement from International Multidisciplinary Roundtable. Med Sci Sports Exerc 2019;51:2375–90. https://doi.org/10.1249/MSS.0000000000002116 31626055 PMC8576825

[10] Schmitz KH, Courneya KS, Matthews C, et al; American College of Sports Medicine. American College of Sports Medicine roundtable on exercise guidelines for cancer survivors. Med Sci Sports Exerc 2010;42:1409–26. https://doi.org/10.1249/MSS.0b013e3181e0c112 20559064

[11] Boyle T, Vallance JK, Ransom EK, et al How sedentary and physically active are breast cancer survivors, and which population subgroups have higher or lower levels of these behaviors? Support Care Cancer 2016;24:2181–90. https://doi.org/10.1007/s00520-015-3011-3 26563180

[12] Coletta AM, Marquez G, Thomas P, et al Clinical factors associated with adherence to aerobic and resistance physical activity guidelines among cancer prevention patients and survivors. PLoS One 2019;14:e0220814. https://doi.org/10.1371/journal.pone.0220814 31369653 PMC6675393

[13] Tarasenko Y, Chen C, Schoenberg N. Self-reported physical activity levels of older cancer survivors: results from the 2014 National Health Interview Survey. J Am Geriatr Soc 2017;65:e39–44. https://doi.org/10.1111/jgs.14589 27943255

[14] Hardcastle SJ, Maxwell-Smith C, Kamarova S, et al Factors influencing non-participation in an exercise program and attitudes towards physical activity amongst cancer survivors. Support Care Cancer 2018;26:1289–95. https://doi.org/10.1007/s00520-017-3952-9 29090387

[15] Ijsbrandy C, Hermens RPMG, Boerboom LWM, et al Implementing physical activity programs for patients with cancer in current practice: patients’ experienced barriers and facilitators. J Cancer Surviv 2019;13:703–12. https://doi.org/10.1007/s11764-019-00789-3 31347009 PMC6828940

[16] Koll TT, Semin JN, Grieb BM, et al Motivating older adults with cancer to keep moving: the implications of lifestyle interventions on physical activity. Curr Oncol Rep 2017;19:68. https://doi.org/10.1007/s11912-017-0623-4 28836159

[17] Ormel HL, van der Schoot GGF, Sluiter WJ, et al Predictors of adherence to exercise interventions during and after cancer treatment: a systematic review. Psychooncology 2018;713:24. 10.1002/pon.4612 PMC5887924 29247584

[18] Lisy K, Kent J, Piper A, et al Facilitators and barriers to shared primary and specialist cancer care: a systematic review. Support Care Cancer 2021;29:85–96. https://doi.org/10.1007/s00520-020-05624-5 32803729

[19] Brandenbarg D, Roorda C, Groenhof F, et al Primary healthcare use during follow-up after curative treatment for colorectal cancer. Eur J Cancer Care 2017;26:e12581. https://doi.org/10.1111/ecc.12581 27726218

[20] Roorda C, Berendsen AJ, Groenhof F, et al Increased primary healthcare utilisation among women with a history of breast cancer. Support Care Cancer 2013;21:941–9. https://doi.org/10.1007/s00520-012-1609-2 23052915

[21] Schaaijk A, Flinterman L, Geit E, et al De praktijkondersteuner huisartsenzorg (POH) in de huisartsenpraktijk: diversiteit en capaciteit. Nivel 2021.

[22] IJsbrandy C, van Harten WH, Gerritsen WR, et al Healthcare professionals’ perspectives of barriers and facilitators in implementing physical activity programmes delivered to cancer survivors in a shared-care model: a qualitative study. Support Care Cancer 2020;28:3429–40. 31792881 10.1007/s00520-019-05108-1 PMC7256088

[23] Neher M, Landén Ludvigsson M, Enblom A. Preparedness to implement physical activity and rehabilitation guidelines in routine primary care cancer rehabilitation: focus group interviews exploring rehabilitation professionals’ perceptions. J Cancer Educ 2021;36:779–86. https://doi.org/10.1007/s13187-020-01704-6 32062799 PMC8328890

[24] Huizinga F, Westerink NDL, Berendsen AJ, et al Implementation and evaluation of a physical activity counselling programme in primary care among cancer survivors: SoDA study protocol. BMJ Open 2022;12:e060098. https://doi.org/10.1136/bmjopen-2021-060098 PMC8896033 35236736

[25] Hwang S, Birken SA, Melvin CL, et al Designs and methods for implementation research: advancing the mission of the CTSA program. J Clin Transl Sci 2020;4:159–67. https://doi.org/10.1017/cts.2020.16 32695483 PMC7348037

[26] Glasgow RE, Harden SM, Gaglio B, et al RE-AIM planning and evaluation framework: adapting to new science and practice with a 20-year review. Front Public Health 2019;7:64. https://doi.org/10.3389/fpubh.2019.00064 30984733 PMC6450067

[27] Gaglio B, Shoup JA, Glasgow RE. The RE-AIM framework: a systematic review of use over time. Am J Public Health 2013;103:e38–46. https://doi.org/10.2105/AJPH.2013.301299 PMC3698732 23597377

[28] Huizinga F, Kieboom EAM, de Greef MHG, et al Cancer survivors’ experiences of a physical activity program in primary care: a qualitative study. J Cancer Surviv 2024. https://doi.org/10.1007/s11764-024-01571-w PMC12460537 38517578

[29] Huizinga F, Westerink N-DL, Walenkamp AME, et al Patient outcomes from a physical activity programme for cancer survivors in general practice: an intervention implementation study. Br J Gen Pract 2025;75:e366. 40044182 10.3399/BJGP.2024.0558 PMC12040372

[30] Twickler R, Berger MY, Groenhof F, et al Data Resource Profile: Registry of electronic health records of general practices in the north of The Netherlands (AHON). Int J Epidemiol 2024;53:dyae021. https://doi.org/10.1093/ije/dyae021 38389286 PMC10884527

[31] Batenburg R, Flinterman L, Vis E, et al Cijfers uit de Nivel-registratie van huisartsen en huisartsenpraktijken. Nivel 2022.

[32] IBM Corp. Released 2015. IBM SPSS Statistics for Windows, Version 23.0. Armonk, NY: IBM Corp.

[33] Braun V, Clarke V. Using thematic analysis in psychology. Qual Res Psychol 2006;3:77–101. https://doi.org/10.1191/1478088706qp063oa

[34] Damschroder LJ, Reardon CM, Widerquist MAO, et al The updated consolidated framework for implementation research based on user feedback. Implement Sci 2022;17:75. https://doi.org/10.1186/s13012-022-01245-0 36309746 PMC9617234

[35] Chan RJ, Crawford-Williams F, Crichton M, et al Effectiveness and implementation of models of cancer survivorship care: an overview of systematic reviews. J Cancer Surviv 2023;17:197–221. https://doi.org/10.1007/s11764-021-01128-1 34786652 PMC8594645

[36] Chan RJ, Agbejule OA, Yates PM, et al Outcomes of cancer survivorship education and training for primary care providers: a systematic review. J Cancer Surviv 2022;16:279–302. https://doi.org/10.1007/s11764-021-01018-6 33763806 PMC7990618

[37] Gyldenvang HH, Piil K, Dahl TH, et al Changing an exercise behaviour for physically inactive patients with breast cancer during chemotherapy- the critical role of nurse support: an implementation study. Eur J Oncol Nurs 2025;74:102807. https://doi.org/10.1016/j.ejon.2025.102807 39874711

[38] Capozzi LC, Daun JT, Francis GJ, et al Feasibility and implementation of an oncology rehabilitation triage clinic: assessing rehabilitation, exercise need, and triage pathways within the Alberta Cancer Exercise-Neuro-Oncology Study. Curr Oncol 2023;30:6220–45. https://doi.org/10.3390/curroncol30070461 37504321 PMC10377964

[39] Brandenbarg D, Maass SWMC, Geerse OP, et al A systematic review on the prevalence of symptoms of depression, anxiety and distress in long-term cancer survivors: implications for primary care. Eur J Cancer Care (Engl) 2019;28:e13086. https://doi.org/10.1111/ecc.13086 31087398 PMC9286037

[40] Maass S, Brandenbarg D, Boerman LM, et al Fatigue among long-term breast cancer survivors: a controlled cross-sectional study. Cancers 2021;13:1301. https://doi.org/ https://doi.org/10.3390/cancers13061301 33803966 PMC8001130

[41] Dossett LA, Hudson JN, Morris AM, et al The primary care provider (PCP)-cancer specialist relationship: a systematic review and mixed-methods meta-synthesis. CA Cancer J Clin 2017;67:156–69. https://doi.org/10.3322/caac.21385 27727446 PMC5342924

[42] Mitchell GK, Burridge LH, Colquist SP, et al General practitioners’ perceptions of their role in cancer care and factors which influence this role. Health Soc Care Community 2012;20:607–16. https://doi.org/10.1111/j.1365-2524.2012.01075.x 22804847

[43] Morgan MA. Cancer survivorship: history, quality-of-life issues, and the evolving multidisciplinary approach to implementation of cancer survivorship care plans. Oncol Nurs Forum 2009;36:429–36. https://doi.org/10.1188/09.ONF.429-436 19581233

[44] Bryant J, Boyes A, Jones K, et al Examining and addressing evidence-practice gaps in cancer care: a systematic review. Implement Sci 2014;9:37. https://doi.org/10.1186/1748-5908-9-37 24666544 PMC4114221

