# Supplementary material for: Mixed-methods organizational evaluation of a physical activity programme for cancer survivors in primary care
Source: Transl Behav Med. 2025 Jul 7;15(1):ibaf029. doi: 10.1093/tbm/ibaf029 (PMC12230946; doi:10.1093/tbm/ibaf029)
Supplement: ibaf029_suppl_Supplementary [file ibaf029_suppl_supplementary.docx]

**Supplement 1.** Topic Lists for Interviews of GPs and PCPs

**Topic List for Interviews of GPs**

| Implementation |
| --- |
| What was the reason you, as a general practice, decided to implement this PA programme? |
| What was your role as a GP in the implementation of this PA programme? |
| Which colleagues were further involved and what was their role in the implementation? |
| How did you experience the implementation of this PA programme? |
| To what extent was the programme feasible for you to give? |
| What helped you in implementing this PA programme? |
| What were barriers or drawbacks you encountered in implementing this programme? |
| Maintenance |
| What would help to make such a PA programme standard GP care? |
| To what extent does this PA programme add to the regular care for cancer survivors from general practice? |
| How do you think care for cancer survivors should be organized in general practice? |

**Topic List for Focus Group or Interviews of PCPs**

| Implementation |
| --- |
| What was the reason you, as a general practice, decided to implement this PA programme? |
| What are your experiences in offering the PA programme to patients? |
| To what extent was the programme feasible for you to give? |
| What helped you in implementing this PA programme? |
| What were barriers or drawbacks you encountered in implementing this programme? |
| Which colleagues were further involved besides you as a PCP and what was their role in the implementation? |
| To what extent does this PA programme suits or adds to your work as PCP? |
| Maintenance |
| What would help to make such a PA programme standard GP care? |
| To what extent does this PA programme add to the regular care for cancer survivors from general practice? |
| How do you think care for cancer survivors should be organized in general practice? |

**Abbreviations:** GP, general practitioner; PA, physical activity; PCP, primary care practitioner (i.e., a practice nurse, dietician, or doctor’s assistant)

| **General Practice** | **PCP or GP** | **Age (years)** | **Sex (M/F)** | **Working Experience (years)** | **Affinity Lifestyle Care (1–5)** | **Affinity Oncology (1–5)** | **Interview Type** |
| --- | --- | --- | --- | --- | --- | --- | --- |
| **1** | PN1 | 41 | F | 10 | 4 | 4 | Focus group |
|  | GP1a | 47 | F | 17 | 3 | 2 | Individual |
|  | GP1b | 49 | F | 18 | 4 | 4 | Individual |
| **2** | PN2 | 49 | F | 15 | 5 | 4 | Focus group & duo |
|  | GP2 | 64 | M | 30 | 4 | 4 | Duo |
| **3** | PN3 | 51 | F | 3 | 5 | 4 | Focus group & individual |
| **4** | DT4 | 50 | F | 27 | 5 | 4 | Focus group & duo |
|  | GP4 | 47 | F | 13 | 5 | 4 | Duo |
| **5** | PN5a | 38 | F | 11 | 5 | 2 | Focus group & duo |
|  | PN5b | 51 | F | 3 | 4 | 4 | Focus group |
|  | GP5 | 33 | M | 4 | 4 | 3 | Duo |
| **6** | PN6 | 56 | F | 18 | 5 | 2 | NA |
|  | GP6 | 36 | F | 8 | 5 | 4 | Individual |
| **7** | PN7 | 41 | F | 5 | 5 | 5 | Focus group |
| **8** | PN8a | 55 | F | 11 | 5 | 2 | Duo* |
|  | PN8b | NA | F | NA | NA | NA | NA |
|  | GP8 | 53 | M | 22 | 1 | 3 | Duo* |
| **9** | DA9 | 40 | F | 20 | 5 | 4 | Duo |
|  | GP9 | 62 | F | 30 | 5 | 3 | Duo |
| **10** | PN10 | 63 | F | 12 | 5 | 4 | Duo |
|  | GP10 | 39 | F | 9 | 4 | 4 | Duo |
| **11** | PN11 | 58 | F | 16 | 5 | 1 | Individual* |
| **12** | PN12 | 48 | F | 9 | 5 | 4 | Individual |
| **13** | PN13 | 47 | F | 2 | 4 | 1 | Duo |
|  | GP13 | 56 | M | 21 | 4 | 5 | Duo |
| **14** | DA14 | 59 | F | 2 | 4 | 4 | NA |
|  | GP14 | 45 | M | 12 | 4 | 3 | Individual* |

**Supplement 2.** Characteristics of PCPs and GPs

Abbreviations: DA, doctor’s assistant; DT, dietician; GP, general practitioner; PCP, primary care practitioner; PN, practice nurse.

* interviews were not recorded; instead, written notes afterwards were used as input
